# Adenofibroma of Skene's Duct: A Case Report

**DOI:** 10.4061/2010/318973

**Published:** 2010-04-28

**Authors:** Yosep Chong, Minseob Eom, Kwang Hwa Park, Hyun Chul Chung, Jae Y. Ro

**Affiliations:** ^1^Department of Pathology, Wonju College of Medicine, Yonsei University, 162 Ilsan-dong, Wonju, Gangwon-do 220-701, South Korea; ^2^Department of Urology, Wonju College of Medicine, Yonsei University, 162 Ilsan-dong, Wonju, Gangwon-do 220-701, South Korea; ^3^Department of Pathology, Weill Medical College, The Methodist Hospital, Cornell University, 6565 Fannin Street, Houston, TX 77030, USA

## Abstract

Skene's glands, also known as paraurethral glands, are homologues of the male prostate, in which painless cystic masses and inflammation due to obstruction have been rarely found and reported. In addition, there have been rare reported cases of adenocarcinoma of Skene's glands. Recently, the authors experienced the first case of adenofibroma arising in Skene's glands of a 62-year-old woman with coital pain. Hereby, we present the case with pathologic and immunohistochemical findings and a short review of literature.

## 1. Introduction

Skene's glands and ducts are one of thirty normal duct structures in the female genital tract which were first introduced and described by Alexander Johnston Chalmers Skene in 1880. These have been thought to be homologues of the male prostate. A pair of glands is located posterolaterally to the urethra and opens its opening at the end of the urethra. Histologically, it is composed of glandular structures which are lined by pseudostratified columnar epithelium and ductal structures which are lined by either stratified squamous or transitional epithelium. Skene's glands secrete a small amount of mucoid material for sexual stimulation. A few kinds of diseases such as painless cystic mass and inflammation due to ductal obstruction were rarely documented [1–3]. In addition, there have been several case reports on adenocarcinoma arising from Skene's glands [4]. 

 Adenofibroma is a mixed biphasic tumor with glandular and fibrous proliferation. Most adenofibromas have been reported from ovary, breast, and uterine cervix. Rare cases of testicular and endometrial adenofibromas have been reported, but to the best of our knowledge, no cases have been reported from Skene's glands. Recently, we experienced a case of adenofibroma arising in Skene's glands of a 62-year-old woman with coital pain. Hereby, we present the first such case of adenofibroma of Skene's glands with pathologic and immunohistochemical findings and a short review of literature.

## 2. Case Report

A 62-year-old woman presented to our hospital with a red erythematous tumor in the external genitalia and coital pain. She had no specific past or family history and did not show any other abnormality except the tumor on physical examination. An excision on that lesion was performed with the clinical impression of urethral caruncle. Serum prostatic specific antigen (PSA) was not measured preoperatively.

**Figure 1:**
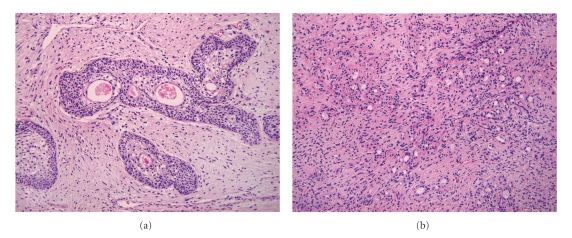
Microscopic findings. (a) Atypical glandular structures including mucinous materials in the lumens are irregularly dispersed in the fibrous stroma. Epithelium lining these glandular structures shows urothelial differentiation with focal mucinous metaplasia. (b) In another area, there is extensive proliferation of both small glandular structures and admixed fibroblasts.

**Figure 2:**
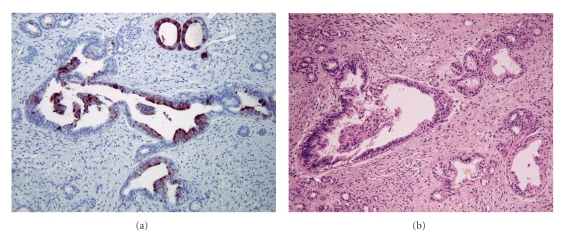
Immunohistochemical stain for prostate specific antigen (PSA). Epithelium of the glandular structures shows strong positivity for PSA (a). Corresponding H&E is seen in (b).

Grossly, the tumor was of a reddish tan ovoid mass, measuring 1.2 × 1 × 1 cm, covered by pale tan mucosa with focal increased vascularity and showing gray white homogenous fibrotic cut surface. Microscopically, the external surface was covered by stratified squamous epithelium and the inner part of the tumor revealed a moderate proliferation of two kinds of atypical glandular structures, relatively large glands containing mucoid material and tiny glands dispersing irregularly (Figures 1(a) and 1(b)). There was diffuse proliferation of fibroblasts around glandular structures. The larger glandular structures were mostly lined by pseudostratified urothelial epithelium with occasional mucinous metaplasia. Although mitoses were not identified, some parts of the glands revealed moderate to high degree of cellular atypia. On immunohistochemistry, some of these glandular structures revealed positivity for PSA (DAKO, Denmark, 1 : 40) (Figure 2(a)). Collectively, the tumor had been diagnosed as an adenofibroma arising in Skene's glands.

On follow-up physical examination after the surgery, the patient did not complaint about coital pain anymore.

## 3. Discussion

Adenofibroma is a term which designates a benign biphasic tumor composed of atypical proliferation of glandular epithelium and surrounding fibrous stromal cells. Most of adenofibroma have been reported from the ovary and breast. A few cases in the uterine endometrium [5], cervix [6], rete testis [7], and paratesticles [8] have been described in the literature. Despite extensive search of the literature, there has been no case report of adenofibroma arising in Skene's glands. 

Microscopically, the lesion described herein could be best classified as an example of adenofibroma with glandular and fibrous stromal proliferation. Because the glandular structures were lined by transitional type cells, urethral caruncle was the main differential diagnosis. However, because of lack of vascular dilatation and inflammatory background, urethral caruncle was easily excluded. Since the lesion was located at the paraurethral area, the possibility of Skene's gland origin lesion was raised. This case was unique for it has been confirmed with the immunohistochemical stain for PSA. 

When patients present with paraurethral mass, the main clinical differential diagnoses include urethral caruncle, cyst, inflammatory abscess, and a possibility of malignant tumor. In our case, the main pathologic differential diagnoses included adenofibroma, urethral caruncle, and less likely an adenocarcinoma with stromal reaction. Based on the lack of inflammatory and vascular changes as well as obvious malignant cytohistomorphologic changes, the possibility of caruncle and carcinoma was excluded. The reason why there have been no reported cases of adenofibroma in this location is a possibility that adenofibromas of Skene's glands could be misdiagnosed as urethral caruncle, clinically and pathologically.

 To find out the etiology or pathogenesis of this disease, however, further studies on this disease might be needed. When we face lesions from the paraurethral locations, besides of common cystic lesions or abscesses, the possibility of adenofibroma should be considered with doing PSA immunostaining.
